# Simplified Assay for Epigenetic Age Estimation in Whole Blood of Adults

**DOI:** 10.3389/fgene.2016.00126

**Published:** 2016-07-14

**Authors:** Laura Vidal-Bralo, Yolanda Lopez-Golan, Antonio Gonzalez

**Affiliations:** Laboratorio de Investigacion 10 and Rheumatology Unit, Instituto de Investigacion Sanitaria – Hospital Clinico Universitario de SantiagoSantiago de Compostela, Spain

**Keywords:** epigenetic, biological age, blood, DNA methylation, MS-SnuPE, biomarker

## Abstract

Biological age is not always concordant with chronological age and the departures are of interest for understanding how diseases and environmental insults affect tissue function, organismal health, and life expectancy. The best-known biological age biomarker is telomere length, but there are more accurate biomarkers as the recently developed based in epigenetic, transcriptomic, or biochemical changes. The most accurate are the epigenetic biomarkers based on specific changes in DNA methylation referred as DNA methylation age measures (DmAM). Here, we have developed and validated a new DmAM that addresses some limitations of the previously available. The new DmAM includes the study in whole blood (WB) of 8 CpG sites selected as the most informative on a training set of 390 healthy subjects. The 8 CpG DmAM showed better accuracy than other DmAM based in few CpG in an independent validation set of 335 subjects. Results were not significantly influenced by sex, smoking, or variation in blood cell subpopulations. In addition, the new 8 CpG DmAM was amenable to study in a single multiplex reaction done with methylation-sensitive single-nucleotide primer extension (MS-SNuPE), a methodology based on commercially available reagents and run in capillary electrophoresis sequencers. In this way, the high cost of DNA methylation microarrays or of a pyrosequencer, which are needed for alternative DmAM, was avoided. Performance of the DmAM with MS-SNuPE was assessed in a set of 557 donors, showing high call rate (>97%), low CV (<3.3%) and high accuracy (Mean Absolute Deviation = 6.07 years). Therefore, the 8 CpG DmAM is a feasible and accurate tool for assessing the epigenetic component of biological age in blood of adults.

## Introduction

Biomarkers of biological age are very useful for identifying situations of premature aging (Lopez-Otin et al., 2013). The best-known biomarker of this type is telomere length, which is shortened during each cell division in cells lacking the telomerase enzyme (Oeseburg et al., 2010; Zhu et al., 2011). It has been found prematurely shortened in blood cells of patients with a variety of diseases and in relation with decreased life expectancy. The appeal of this technology is such that several companies have been created to offer predictions of life expectancy and of health quality based in telomere length analyses (Leslie, 2011; Wolinsky, 2011). However, biological aging is a complex process and telomere length is unable to inform of all its aspects, making it an inaccurate biomarker in many instances. Recently, several new biomarkers of age of increased accuracy and content have been developed. One of these new biomarkers is made by combining the levels of multiple biochemical routine blood tests (Putin et al., 2016), others include information on the expression of hundreds of genes (Peters et al., 2015). The most accurate of all them are the epigenetic biomarkers (Weidner et al., 2014; Peters et al., 2015; Putin et al., 2016), which have become possible after the identification of age associated changes in DNA methylation at specific CpG sites (Fraga et al., 2005). The mechanism of these age-related changes is incompletely understood. Most experiments suggest that it involves perturbations of the DNA methylation maintenance system that lead to slowly accumulating failures along time (epigenetic drift; Hannum et al., 2013; Horvath, 2013; Teschendorff et al., 2013). The same experiments also indicate that epigenetic drift can be accelerated by somatic mutations, cell divisions, and environmental stress. The biomarkers based on these changes are referred as DNA methylation age measures (DmAM) or epigenetic clocks (Bocklandt et al., 2011; Hannum et al., 2013; Horvath, 2013; Florath et al., 2014; Weidner et al., 2014; Huang et al., 2015; Zbiec-Piekarska et al., 2015). These biomarkers combine information from several CpG sites that experience either increased or decreased methylation with age. Some of them were developed for a single tissue (Bocklandt et al., 2011; Florath et al., 2014; Weidner et al., 2014; Huang et al., 2015; Zbiec-Piekarska et al., 2015), most often blood, and others were developed and validated for multiple tissues (Hannum et al., 2013; Horvath, 2013). The DmAM in blood show good correlation with chronological age (Hannum et al., 2013; Horvath, 2013; Florath et al., 2014; Weidner et al., 2014; Huang et al., 2015; Zbiec-Piekarska et al., 2015), which is better than the obtained with telomere length (Weidner et al., 2014) and with other biological age biomarkers (Peters et al., 2015; Putin et al., 2016). In addition, the DmAM in blood show accelerated aging in progressive bone marrow failure syndromes (Weidner et al., 2014) and in Down syndrome (Horvath et al., 2015a), as well as, correlation with cognitive and physical fitness in the elderly (Marioni et al., 2015b), and with all-cause mortality in aged subjects (Marioni et al., 2015a; Christiansen et al., 2016), or the opposed association with familiar longevity (Horvath et al., 2015b). Fulfilling, therefore, all the characteristics of an accurate biomarker of biological age, useful to study how its departures from chronological age affect tissue function, organismal health and life expectancy.

Some DmAM use methylation at a large number of CpG sites (Hannum et al., 2013; Horvath, 2013), requiring whole genome methylation arrays. However, this is a too expensive technology for studies aiming to analyze biological age in a large number of samples. Alternatives with fewer CpG sites are already available for studies of saliva, 2 or 3 sites (Bocklandt et al., 2011), and WB, from 3 to 17 sites (Florath et al., 2014; Weidner et al., 2014; Huang et al., 2015; Zbiec-Piekarska et al., 2015). They still could be problematic in some settings because they require a pyrosequencer and this equipment is not available in many laboratories. In addition, the available DmAM were developed including the whole range of ages, from birth to very old age, and this is not possible without losing accuracy because the rate of changes is faster in pre-adolescents than in adults and follows different dynamics, exponential vs. lineal (Alisch et al., 2012; Horvath, 2013). Therefore, we aimed to develop and validate a simplified DmAM with the following characteristics: using WB, requiring a single reaction per patient, calibrated for adults, and amenable to focused analysis of a few CpG sites in laboratories lacking a pyrosequencer. The technology used involves methylation-sensitive single-nucleotide primer extension (MS-SNuPE; Kaminsky et al., 2005), which is based in commercially available reagents and requires a capillary sequencer.

## Materials and Methods

### DNA Methylation Data Sets

We used four sets of blood cell DNA methylation data (**Table 1**). One was used for development of the DmAM and was named training set. This training set included data from the 390 donors older than 20 years from Weidner et al. (2014), which were obtained with the Illumina Human Methylation 27K BeadChip platform and are available under GSE19711, GSE20242, GSE20236, GSE23638 GEO accession numbers. The next three data collections (**Table 1**) were validation sets used to assess different aspects of the new DmAM. The first was from Liu et al. (2013). It was used to compare the accuracy of the new DmAM with previous DmAM. This data set includes Illumina HumanMethylation 450K BeadChip information obtained from 335 donors recruited randomly from the Swedish national population registry and publicly available with GSE42861 accession number. The second validation data set was used to evaluate the effect of heterogeneity in blood cell composition on the new DmAM. It included DNA methylation and blood cell composition from 92 individuals in the Vancouver lower mainland area, who were studied by Lam et al. (2012). Methylation data were obtained with the Illumina Human Methylation 27K BeadChip and they are available under the GSE37008 accession number. Blood cell composition included the number of monocytes, lymphocytes, neutrophils, basophils, and eosinophils assessed with an Advia 70 (Siemens Medical) system. Samples from these previous studies were according with ethical requirements as reported in the primary publications (Lam et al., 2012; Liu et al., 2013; Weidner et al., 2014). The third validation data set was obtained for this study. It was used to test the performance of the new DmAM on DNA methylation data obtained with MS-SNuPE. It included methylation data from DNA samples of 557 donors of European Spanish ancestry. These subjects were recruited as controls for studies of rheumatic diseases during ambulatory explorations. Most of them, 375, were recruited during preoperative work-up for elective minor surgeries other than joint surgery. The remaining 182 subjects were recruited during intravenous urography. Patients with bad health status (physical or mental) or with symptoms or signs of OA and patients with inflammatory or autoimmune diseases, as well as those reporting foreign ancestors were excluded. The Ethics Committee for Clinical Research of Galicia approved study of this third validation set for which all participants have given written informed consent.

**Table 1:** Detailed description of the sample collections used in this study.

Application	Study	*N*	Age *(SD)*	Age range	Woman%
Training	Weidner et al., 2014	390	61.2 (11.6)	20–78	96.7
DNA methylation age measures (DmAM) comparison	Liu et al., 2013	335	52.8 (11.5)	20–70	71.3
Blood composition	Lam et al., 2012	92	52.8 (11.5)	25–45	71.3
MS-SNuPE validation	Current study	557	65.9 (10.0)	45–89	51.4

*N = Sample size; Age = mean age in years; *SD* = Standard deviation.*

### Definition of DmAM Based on 8 CpGs

We used the 390 healthy Caucasian donors older than 20 years from Weidner et al. (2014), to define a DmAM optimized to estimate age from blood DNA in adult subjects and allowing assays with MS-SNuPE. The dataset contains DNA methylation profiles of 102 CpGs strongly correlated with age (Pearson correlation coefficient *r* > 0.85 or *r* < –0.85). We selected the most informative by forward stepwise linear regression. At each step, feasibility of assay by MS-SNuPE of the CpG entering the model was checked. If the assay was possible, the CpG was incorporated to the regression model, on the contrary, it was discarded and the linear regression restarted without it. A total of 8 CpGs were incorporated to the model with a 0.05 P threshold to enter. The 8 CpGs and the B coefficient values obtained with multiple linear regression on the training set are detailed in **Table 2**. The 8 CpG DmAM was further evaluated for accuracy with the training set and the three validation data sets. The first validation set was specifically used for comparing the 8 CpG DmAM obtained with three other DmAM (Hannum et al., 2013; Horvath, 2013; Weidner et al., 2014), because this data set included blood DNA methylation data that have not been used to calibrate any of the DmAM. The role of gender and smoking was analyzed by multiple regression against the difference between age and the DmAM estimation. The DmAM used for comparison were selected because the availability of the model parameters and of the methylation information at the required CpG sites. It should be noted that the Hannum DmAM was used without clinical variables because of lack of the necessary information (Hannum et al., 2013). Accuracy of DmAM was assessed as correlation with chronological age, and as mean difference and mean absolute deviation (MAD) between predicted age and chronological age. In the analysis of blood cell subpopulation, cell counts were *Z* transformed for representation (except for basophils, which only showed 0 or 1 counts per 10 μl). These analyses were done with Statistica 7.0 (Stat Soft, Inc.).

**Table 2:** Multiple linear regression parameters of the 8 CpG DmAM.

Term	*B*	*SE*	*t*-value	*p*-level
Intercept	84.7	4.3	19.5	<1.0 × 10^-16^
cg16386080	59.5	4.9	12.3	2.4 × 10^-29^
cg24768561	33.9	5.9	5.8	1.5 × 10^-08^
cg19761273	–44.0	9.8	4.48	1.0 × 10^-05^
cg25809905	–19.7	5.4	3.7	2.9 × 10^-04^
cg09809672	–22.8	6.5	3.5	5.0 × 10^-04^
cg02228185	–16.8	4.8	3.5	5.5 × 10^-04^
cg17471102	–17.7	6.5	2.7	0.006
cg10917602	–11.4	5.1	2.2	0.026

*This model was calibrated with the training data set. *SE* = Standard Error.*

### Assays of DNA Methylation with MS-SNuPE

Genomic DNA, 1 μg, was bisulfite-converted in 96 deep-well Methylation-Gold kit plates (Zymo Research, USA) following the manufacturer specifications. Oligonucleotide primers and probes for multiplex Ms-SNuPE reactions assaying the 8 selected CpGs were designed with MethPrimer (Li and Dahiya, 2002) and are provided in Supplementary Table S1. Multiplex Ms-SNuPE reactions were performed as previously described (Kaminsky et al., 2005). Optimal amounts of primers and probes were determined to avoid saturation or inefficient reaction. Reactions without bisulfite-converted DNA were included to assess specificity. All samples were assayed in duplicate and those with CV higher than 10% were repeated. A control sample was run in all plates.

## Results

### New DmAM Amenable for MS-SNuPE and Calibrated for Adults

**FIGURE 1:**
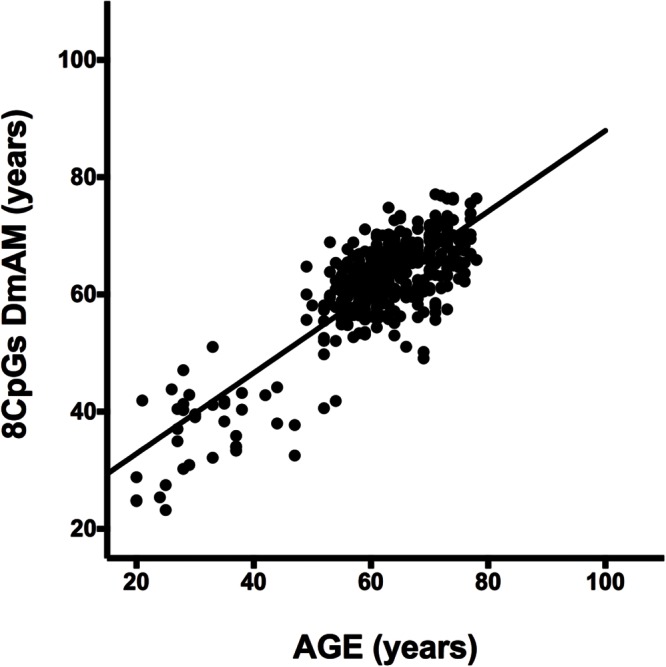
**Results of the 8 CpG DNA methylation age measures (DmAM) in the training set.** The scatterplot represents age in the horizontal axis, against the 8 CpG DmAM in the vertical axis from the healthy donors of the training set (*n* = 390). Straight lines represent least squares regression fit to the data.

We selected CpG sites for the new DmAM among the 120 CpGs with stronger correlation by forward stepwise linear regression with age in Weidner et al. (2014). For this process, we used the training set of 390 healthy subjects older than 20 years of age. At each step, we checked the CpGs for their compatibility with MS-SNuPE assays (Supplementary Table S1). The process finished with 8 CpGs, each of them showing significant contribution in multiple regression (**Table 2**), with 2 showing an increase in methylation with age and 6 showing a decrease. The new 8 CpG DmAM provided an accurate estimation of age in the training set (*R*^2^ = 0.68, *P* < 10^-16^; MAD = 5.07 years; **Figure 1**). In addition, accuracy was similar in different age categories from 30 to 80 years of age (**Table 3**). The lower age group, below 30 years of age, showed a larger MAD and larger difference in mean than the other strata (**Table 3**). It is worth to note that the highest accuracy is observed near the mean age of the training set. This is common to all predictions based on regression.

**Table 3:** Accuracy of 8 CpG DmAM in different age intervals.

Age interval	Mean age	*N*	MAD	Mean difference
20–29	25.4	17	9.2	9.0
30–49	39.5	23	6.6	3.1
50–59	56.3	93	4.9	3.6
60–69	64.0	158	4.1	–0.3
70–78	72.8	99	5.7	–5.2
All	61.2	390	5.1	0.00

*N = Sample size, MAD = Mean absolute deviation.*

### Relative Accuracy of the New DmAM

We used the first validation set to assess the relative accuracy of the 8 CpG DmAM in relation with three other DmAMs (Hannum et al., 2013; Horvath, 2013; Weidner et al., 2014). This validation set includes blood DNA methylation data from 335 healthy adults (Liu et al., 2013). The best models were the two based in a large number of CpG and a sophisticated prediction model (**Table 4**). They showed the lowest MAD and the strongest correlation with chronological age. The DmAM of Horvath excelled in MAD and mean difference, whereas the DmAM of Hannum showed the strongest correlation with chronological age. It is important to note that the Hannum DmAM was used only with methylation data, without the clinical data included in its original description (Hannum et al., 2013).

**Table 4:** Comparative performance of the 8 CpG DmAM with other DmAM in the 335 blood samples from the first validation set.

DmAM	N° CpGs	MAD	Adjusted *R*^2^	Spearman rho	Mean difference
Horvath, 2013	353	4.4	0.77	0.87	–1.0
Hannum et al., 2013	71	7.1	0.84	0.90	–6.7
8 CpGs	8	7.3	0.60	0.75	–4.8
Weidner et al., 2014	3	8.5	0.33	0.57	3.8

*MAD = Mean absolute deviation.*

The new 8 CpG DmAM showed an accuracy that was intermediate between the DmAM based in many CpGs and the based in few. It was nearer to any of the two best DmAM than to the Weidner DmAM in the correlation coefficients. In addition, it showed a lower mean difference between age and the DmAM estimation than the Hannum DmAM and a comparable MAD. Therefore, the 8 CpG DmAM was more accurate than the Weidner DmAM, but less accurate than the methods requiring microarray analysis of DNA methylation. These results indicate that the 8 CpG DmAM provides an improved compromise between feasibility and accuracy.

### The New 8 CpGs DmAM Was Independent of Heterogeneity in Blood Cell Counts and Other Confounding Factors

**FIGURE 2:**
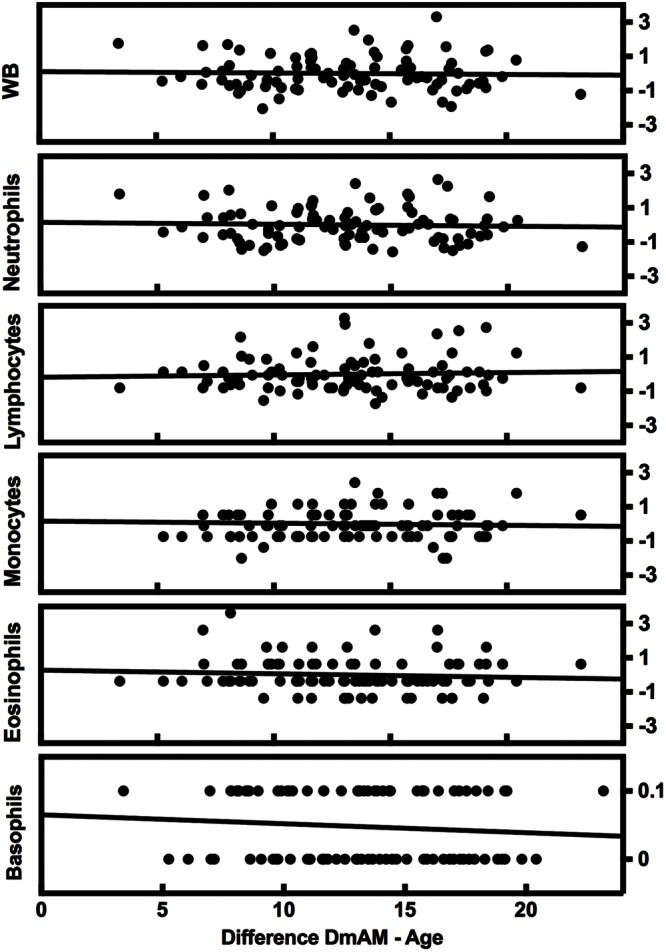
**Lack of variation of the 8 CpG DmAM with changes in blood cell composition from the second validation set.** Each rectangle represent the linear regression of (8 CpGs DmAM – age) against the *Z* transformed cell counts of whole blood (WB) cells, neutrophils, lymphocytes, monocytes, and eosinophils, respectively. Basophil counts were not transformed due to their dichotomous distribution.

The complexity of blood composition and its variability has been mentioned as a possible confounding factor for DmAM. Therefore, we checked the influence of changes in blood cell counts on the new 8 CpG DmAM using the second validation set (Lam et al., 2012). No association was found with cell counts of any of the major subpopulation or with variation in WB cell numbers (**Figure 2**). All the β coefficients were <0.03 with *P*-values >0.6 (Supplementary Table S2). In contrast, association with age showed a β coefficient of 0.77 with *P* < 10^-17^.

Other possible confounding factors, sex and smoking were available in the first validation set. They were analyzed in this set because of the higher power of analysis in the 335 subjects on it (Liu et al., 2013). The 8 CpG DmAM was neither associated with smoking (β = –0.020, *P* = 0.6), nor with sex (β = –0.005, *P* = 0.9).

### Validation of the 8 CpG DmAM with MS-SNuPE

**FIGURE 3:**
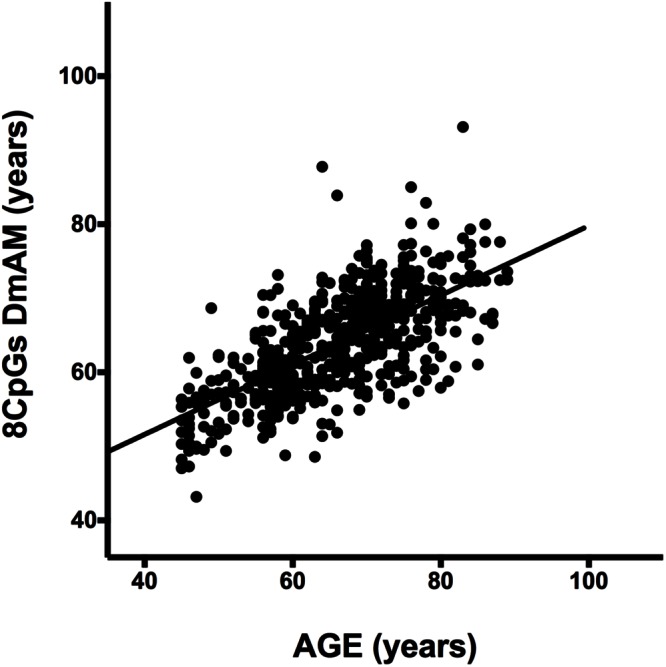
**Scatterplot of age against the 8 CpG DmAM from the healthy donors of the third validation set analyzed with MS-SNuPE (*n* = 557).** Straight lines represent least squares regression fit to the data.

We tested accuracy of the new 8 CpG DmAM in the third set of samples, in which the 8 CpG methylation levels were determined by MS-SNuPE for the current study. The MS-SNuPE assay showed a 97.6% call rate, and a between plates reproducibility of 3.3% CV. The 8 CpG DmAM showed good accuracy in relation with the observed in other sample sets with this same DmAM and observed with other DmAM in the first validation set. The MAD and the mean difference (MAD = 6.07 years, mean difference = –2.1 years) were better than the observed in the first validation set with the same DmAM (**Table 4**). These differences were only slightly larger than the observed in the training set used to define the model parameters. Linear correlation with chronological age, in contrast, was weaker than the observed in the first validation set (adjusted *R*^2^ = 0.45, *P* < 10^-16^; Spearman *R* = 0.67). However, the distribution of values was concentrated around the regression line with only some subjects showing wide differences between chronological age and the age estimated with the DmAM (**Figure 3**). This indicates that the decrease in correlation coefficient was related with the smaller range of ages in the third validation set (age range = 35 years, vs. 50 years in the first validation set) for an even smaller residual standard deviation (7.4 years vs. 8.2 years).

## Discussion

We have developed a new DmAM that is appropriate for large studies done with blood samples of adult subjects in laboratories counting with a capillary sequencer. This DmAM has reached an improved compromise between feasibility and accuracy. It allows detection of changes in epigenetic aging with accuracy slightly lower than DmAM that are much more costly per sample because they require whole genome DNA methylation microarrays (Hannum et al., 2013; Horvath, 2013). In addition, it provides results that are independent of important confounders and easily comparable with the obtained with other technologies.

The DNA methylation microarrays provide a large wealth of information that could be of interest for other analyses, but their cost is excessive if the objective is only to assess epigenetic age. This consideration has led to the development of DmAM with few CpG sites for large epidemiological studies, and forensic applications (Bocklandt et al., 2011; Florath et al., 2014; Weidner et al., 2014; Huang et al., 2015; Zbiec-Piekarska et al., 2015). Among the DmAM requiring few CpG sites, the new 8 CpG DmAM showed better accuracy in our analyses than other DmAM developed also for blood samples (Weidner et al., 2014). Unfortunately, a direct comparison with the other DmAM in this category was impossible because of lack of the necessary information, either because the CpG sites are not in DNA microarrays or because the model parameters were not reported (Florath et al., 2014; Huang et al., 2015; Zbiec-Piekarska et al., 2015). Independently of their accuracy, which we will further consider below, all the other DmAM based on few CpG are designed for assay by pyrosequencing, which is not available in many laboratories. The accuracy of this type of DmAM increases with the inclusion of additional CpGs (Bocklandt et al., 2011; Weidner et al., 2014), but each new CpG requires a new reaction with its associated cost and time. In contrast, the MS-SNuPE technology has the advantage of its multiplexing nature allowing the analysis of the 8 CpG DmAM in a single reaction reducing the expenses and the time needed (Kaminsky et al., 2005). We have estimated that the 8 CpG DmAM will require about 32 h after bisulfite modification whereas the MS-SNuPE needs 8 h in equipment that is widely available: a PCR thermocycler and a capillary sequencer.

It is important to note that accuracy of any of the DmAM is partly age dependent, and this dependence has two components. The first is due to the difference between the training set mean age and the age to be estimated. The best age estimations are obtained near the mean value of the training set, because the regression parameters are estimated with this set. This dependence means that regression parameters show bias in other sample collections different from the training set. The second component is due to the range of ages considered. This range affects correlation coefficients as measures of accuracy. Because of the correlation coefficient formula, a wide range of ages leads to higher correlation coefficients than a narrow range for the same dispersion of data around the regression line. In addition, the range of ages affects accuracy of the estimation due to the different dynamics of DNA methylation changes with age in children and in adults, as already mentioned (Alisch et al., 2012; Horvath, 2013). This variation means that accurate results are difficult with DmAM aiming to cover all age ranges (Horvath, 2013; Zbiec-Piekarska et al., 2015). Therefore, we choose restricting development of a new DmAM to adults. This approach is safer than assuming a constant rate of change of the DmAM for all ages from birth to very old, as has been done by some previous DmAM (Bocklandt et al., 2011; Hannum et al., 2013; Weidner et al., 2014; Huang et al., 2015; Zbiec-Piekarska et al., 2015). An alternative and accurate approach has been followed by the Horvath DmAM, which achieved good accuracy along all age ranges thanks to an elastic net regression model that accounted for a variable rate of change (Horvath, 2013). All the mentioned sources of experiment-specific effects on accuracy of DmAM indicate the need to compare DmAM in the same set of samples, as we have done here.

The previous consideration leads to highlight another good property of the 8 CpGs DmAM. The 8 CpG sites included in the new DmAM are amenable to analysis with any of the common technologies for DNA methylation analysis. They were selected as amenable to MS-SNuPE, among CpG sites available in both Illumina 27K and 450K Bead Chip methylation arrays, and they are amenable to study with pyrosequencing. This characteristic allows a wide comparability with other DmAM in the same data set.

An important consideration for the interpretation of DmAM is their degree of dependence of blood cell composition (Jaffe and Irizarry, 2014). Ideally, the DmAM should be independent of commonly observed changes in blood cell composition. Our analysis showed this independence for the new 8 CpG DmAM in the 92 samples from our second validation sample set (Lam et al., 2012). Some previous DmAM have also demonstrated this property (Horvath, 2013; Weidner et al., 2014). In addition, the 8 CpG DmAM was independent of sex, and smoking habit. This independence could be an advantage over other DmAM that should be adjusted for these confounders (Hannum et al., 2013; Florath et al., 2014).

## Conclusion

We propose a new DmAM for large studies of biological age in blood samples of adults that is amenable to analysis in a single reaction with MS-SNuPE. This DmAM involving 8 CpG sites represents an improvement either in feasibility or in accuracy over previous DmAM.

## Author Contributions

Conception and design the study: LV and AG; Acquisition and analysis of data: YL and LV; Drafting of the article: LV and AG; Critical revision of the article: YL; Final approval of the version to be published: LV, YL, and AG; Agreement to be accountable for all aspects of the work: LV, YL, and AG.

## Conflict of Interest Statement

The authors declare that the research was conducted in the absence of any commercial or financial relationships that could be construed as a potential conflict of interest.
