# Corrigendum: Simplified Assay for Epigenetic Age Estimation in Whole Blood of Adults

**DOI:** 10.3389/fgene.2017.00051

**Published:** 2017-04-21

**Authors:** Laura Vidal-Bralo, Yolanda Lopez-Golan, Antonio Gonzalez

**Affiliations:** Laboratorio Investigacion 10 and Rheumatology Unit, Instituto Investigacion Sanitaria—Hospital Clinico Universitario de SantiagoSantiago de Compostela, Spain

**Keywords:** epigenetic, biological age, blood, DNA methylation, MS-SnuPE, biomarker

In the original article, there was a mistake in Table S1 as published. **Sequences of the primers to amplify 4 of the CpG sites were wrong**. The corrected Table S1 appears below. The authors apologize for this error and state that this does not change the scientific conclusions of the article in any way.

**Table S1:** **CpGs used in the 8 CpG DmAM, the nearby genes, and the primers and probes used to analyze them**.

**CpG**	**Gene**	**Primer forward**	**Primer reverse**	**Probe**
cg09809672	EDARADD	TGAGAAATTTAGGAAGATAGTAAATGTTTA	AATTTATCCTCCCACCTACAAATTCC	TAACCAAACAACCAACIAACATCTTCTC
cg24768561	CENTG2	GTTTTGAGGTAAATGGGATTTT	CCCAACCAATAAACCAACAC	ATAACTAAAAACAAAAACTCAACCAATATCC TCAATCCAAAACCTTATAAAACC
cg16386080	CDK20	TTGGGGTAGGGGATTAAGTTAGTT	TCCCTTTTTACATCCAATACAATTTT	gccagcgtcagacatcatatgcagatacCCAA TACAATTTTTAAAACCTACTCATATTCTAAAC CTACTTTAAACC
cg10917602	HSD3B7	TAGGAAGGTGGGAAGGGT	CATCCCCACCAAATTCTC	gatacCCCTCCAAACCAATCTAAACACCCTA AAATAACIACTACAAATAAACAAAAAC
cg02228185	ASPA	AATTATTTGGTGAAATGATTTTTTGTTATA	AATAATTTTACCTCCAACCCTATTCTCTA	GGAGTATTTTTGGTTAAGTATTGGTTAGA GAATGG
cg25809905	ITGA2B	GGGTTTTGTTTAGGGGAGTTTTT	TTTCCATCCAATCTTTCAACAATAC	attgatcgtggtgatatccgATAAATAATATACTCAAT ACTATACCTACITATATTAACCCAC
cg19761273	CSNK1D	GGAGGTTTTGATGTTTAGTTTGAAG	TCCACTCCTTATTTCCTTTACAAA	AACATTCAAATCCAACACAAATAAAAATATT AACTCCITCTCCAAACC
cg17471102	FUT3	GAAAGATTTTTGTTTGTGATTAGGG	AATTATCCCATTCTACCTTTTCCC	ATAAACCCTAATTCATAATATAA CTAAACTAACACAAAATCCC

*All sequences are in the 5′–3′ direction. Lowercase bases in probes correspond with non-specific tails. Inosines were used for polymorphic positions*.

The original article has been updated.

## Conflict of interest statement

The authors declare that the research was conducted in the absence of any commercial or financial relationships that could be construed as a potential conflict of interest.

