# Supplementary material for: Simplified Assay for Epigenetic Age Estimation in Whole Blood of Adults
Source: Front Genet. 2016 Jul 14;7:126. doi: 10.3389/fgene.2016.00126 (PMC4943959; doi:10.3389/fgene.2016.00126)
Supplement: Supplementary file 1 [file Table_1.DOCX]

Table S1. CpGs used in the 8 CpG DmAM, the nearby genes, and the primers and probes used to analyze them. All sequences are in the 5’ – 3’ direction. Lowercase bases in probes correspond with non-specific tails. Inosines were used for polymorphic positions.

| **CpG** | **Gene** | **Primer Forward** | **Primer Reverse** | **Probe** |
| --- | --- | --- | --- | --- |
| cg09809672 | EDARADD | TGAGAAATTTAGGAAGATAGTAAATGTTTA | AATTTATCCTCCCACCTACAAATTCC | TAACCAAACAACCAACIAACATCTTCTC |
| cg24768561 | CENTG2 | GTTTTGAGGTAAATGGGATTTT | CCCAACCAATAAACCAACAC | ATAACTAAAAACAAAAACTCAACCAATATCCTCAATCCAAAACCTTATAAAACC |
| cg16386080 | CDK20 | TTGGGGTAGGGGATTAAGTTAGTT | TCCCTTTTTACATCCAATACAATTTT | gccagcgtcagacatcatatgcagatacCCAATACAATTTTTAAAACCTACTCATATTCTAAACCTACTTTAAACC |
| cg10917602 | HSD3B7 | TAGGAAGGTGGGAAGGGT | CATCCCCACCAAATTCTC | gatacCCCTCCAAACCAATCTAAACACCCTAAAATAACIACTACAAATAAACAAAAAC |
| cg02228185 | ASPA | AATTATTTGGTGAAATGATTTTTTGTTATA | AATAATTTTACCTCCAACCCTATTCTCTA | GGAGTATTTTTGGTTAAGTATTGGTTAGAGAATGG |
| cg25809905 | ITGA2B | GGGTTTTGTTTAGGGGAGTTTTT | TTTCCATCCAATCTTTCAACAATAC | attgatcgtggtgatatccgATAAATAATATACTCAATACTATACCTACITATATTAACCCAC |
| cg19761273 | CSNK1D | GGAGGTTTTGATGTTTAGTTTGAAG | TCCACTCCTTATTTCCTTTACAAA | AACATTCAAATCCAACACAAATAAAAATATTAACTCCITCTCCAAACC |
| cg17471102 | FUT3 | GAAAGATTTTTGTTTGTGATTAGGG | AATTATCCCATTCTACCTTTTCCC | ATAAACCCTAATTCATAATATAACTAAACTAACACAAAATCCC |
