# Supplementary material for: Simplified Assay for Epigenetic Age Estimation in Whole Blood of Adults
Source: Front Genet. 2016 Jul 14;7:126. doi: 10.3389/fgene.2016.00126 (PMC4943959; doi:10.3389/fgene.2016.00126)
Supplement: Supplementary file 2 [file Table_2.DOCX]

Table S2. Multiple regression analysis between 8 CpG DmAM and cell counts per 10^-9^ liters of blood. Std. Err. = Standard error

|  | Beta | Std.Err. | p-level |
| --- | --- | --- | --- |
| Age | 0.77 | 0.07 | 3.63 x10^-18^ |
| Neutrophils | 0.01 | 0.07 | 0.9 |
| Lymphocytes | -0.02 | 0.07 | 0.7 |
| Monocytes | 0.004 | 0.08 | 0.9 |
| Basophils | 0.03 | 0.08 | 0.7 |
| Eosinophils | 0.03 | 0.07 | 0.7 |
